# Spatial and seasonal patterns of fish assemblages in mountain streams of the Ren River, southwest China

**DOI:** 10.1002/ece3.7917

**Published:** 2021-07-16

**Authors:** Fei Liu, Pengcheng Lin, Huanzhang Liu, Jun Wang

**Affiliations:** ^1^ The Key Laboratory of Biodiversity and Conservation of Aquatic Organisms Institute of Hydrobiology Chinese Academy of Sciences Wuhan China; ^2^ State Key Laboratory of Eco‐hydraulic in Northwest Arid Region of China Xi'an University of Technology Xi'an China

**Keywords:** fish assemblage, fish conservation, mountain stream, spatial–temporal changes

## Abstract

The spatial–temporal patterns of fish assemblages in lotic systems can provide useful information in developing effective conservation measures. This study aimed to explore the spatial and seasonal changes in fish assemblages and their association with environmental factors in mountain streams of the Ren River, southwest China. Field investigations were conducted at 18 sites during the rainy and dry seasons in 2017. A total of 1,330 individuals, belonging to three orders, eight families, 19 genera, and 21 species, were collected. Analysis of similarities (ANOSIM) showed that the structure of fish assemblages varied significantly at the spatial scale, but not at the seasonal scale. In low‐order sites, fish assemblages were mainly dominated by cold‐water and rheophilic species (e.g., *Rhynchocypris oxycephalus*, *Scaphesthes macrolepis*, *Metahomaloptera omeiensis,* and *Gnathopogon herzensteini*), while those in high‐order sites were predominated by warm‐water and eurytopic or stagnophilic species (e.g., *Squalidus argentatus*, *Hemiculter leucisculus,* and *Zacco platypus*). Canonical correspondence analysis (CCA) showed that the fish assemblages were structured by a combination of large‐scale landscape factors (e.g., altitude and C‐link) and small‐scale habitat features (e.g., channel width, water temperature, and depth). Among these factors, landscape had the greatest influence on fish assemblages, while local habitat variables were less important or were only significant in certain seasons.

## Graphical abstract

This study demonstrated how fish assemblages changed at different spatial and temporal scales in this mountain river. Furthermore, environmental factors that contributed most to the observed spatial–temporal patterns of fish assemblage structure were identified. These results provide useful information in understanding the mechanisms structuring fish assemblages and making effective protection measures.

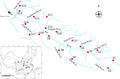

## 1 INTRODUCTION

Understanding how fish assemblages are structured in lotic ecosystems is one of the most important aspects of community ecology and can provide useful information for conservation of biodiversity and ecological restoration (Araújo et al., 2009). The River Continuum Concept (RCC) has suggested that longitudinal variations in the physicochemical environment (e.g., width, depth, velocity, flow volume, and temperature) influence structural and functional characteristics of lotic aquatic communities (Vannote et al., 1980). Species diversity often increases along the upstream–downstream gradient, responding to increasing habitat diversity and food availability (Lowe‐McConnell, 1975). Although the original RCC was developed for aquatic macroinvertebrates, similar patterns have been found for fish assemblages in both temperate and tropical regions (Ferreira & Petrere, 2009; Hughes & Gammon, 1987; Matthews, 1998). Subsequent studies have found that large‐scale landscape position, such as stream order, stream size, link magnitude, confluence link (C‐link), and downstream link (D‐link) are important factors in structuring stream fish assemblages (Fairchild et al., 1998; Fausch et al., 1984; Mullen et al., 2011; Osborne & Wiley, 1992; Smith & Kraft, 2005). For example, Fausch et al. (1984) found fish assemblages changed gradually with stream order and Smith and Kraft (2005) demonstrated that C‐link and stream order were the stream network position measures with the greatest influence on fish assemblages. Osborne and Wiley (1992) observed D‐link explained the greatest portion of the variance in fish species richness and suggested downstream processes significantly influence the structure of fish assemblages. In addition, seasonal variations in environmental conditions caused by periodic flooding are expected to have significant impacts on stream fish assemblages (He et al., 2017; Ostrand & Wilde, 2002). Flooding can increase the diversity and availability of habitat structures and food resources and then lead to changes in fish assemblages between dry and wet seasons (Araújo et al., 2009). Therefore, the current consensus is that stream fish assemblages are structured by a series of local and regional factors operating at multiple spatial and temporal scales (Jackson et al., 2001; Matthews, 1998; Vardakas et al., 2015). However, the relative importance of these factors varies with environmental variability, climate conditions, and nature of fish assemblage in the survey area (Grossman et al., 1998; Jackson et al., 2001).

To date, spatial and temporal variations of stream fish assemblages have been tested in numerous regions, including North America (Mullen et al., 2011; Ostrand & Wilde, 2002), Latin America (Araújo et al., 2009; Fernandes et al., 2013; Habit et al., 2007; Silvano et al., 2000), Europe (Pires et al., 1999; Vardakas et al., 2015), and South Asia (Bhat, 2004). In recent years, similar studies have been also conducted in China. However, nearly all these studies were concentrated in central and eastern regions (He et al., 2017; Li et al., 2012; Yan et al., 2010; Zhu et al., 2017). Southwest China contains a large number of streams and rivers and supports a diverse range of fishes, yet has received little attention.

The Dabashan Mountain is located at the border area of Hubei Province, Chongqing Municipality, Sichuan Province, and Shanxi Province in the southwest China. It is an important water source for many river systems. The Jialing River and the Han River, which represent the largest tributaries of the upper and middle Yangtze River, respectively, both originate from this region. Furthermore, the Dabashan Mountain harbors a high level of biodiversity and species endemicity, which has been listed as one of the 35 biodiversity conservation priority area of China (Ministry of Ecology & Environment of People's Republic of China, 2010). Therefore, the Dabashan Mountain plays important roles in water security and biodiversity conservation. However, little information about the distribution patterns of the fish fauna in this region has been available until now. On the other hand, fish diversity in this region has decreased due to increased human activities, such as overfishing, hydropower station construction, and water pollution (Zeng, 1991). For the development of effective conservation strategies, there is an urgent need to understand the organization mechanisms of fish assemblages in this area.

Therefore, the fish fauna and environmental features in mountain streams of the Ren River, the largest tributary of the upper Han River, were investigated in this study. The main objective was to test whether and how fish assemblages in this mountain river vary at spatial and seasonal scales. Furthermore, we wanted to identify the key environmental factors that contributed most to the observed spatial and seasonal patterns of fish assemblages. We hypothesized that fish assemblages in this mountain river may change significantly spatially but not seasonally, as they are located in the upstream area and dominated mainly by resident fish species. We believe these studies can help us to understand how fish assemblages are structured in such mountain river systems and provide valuable information in future fish conservation and management.

## 2 METHODS

### 2.1 Study area

The Ren River is the largest tributary of the upper Han River and originates in the Dayanshan Mountain in the southern foot of the Dabashan Mountain. It flows for 211.4 km before draining into the Han River in Ziyang County, Shanxi Province, with a drainage basin of 4,871 km^2^. The whole basin is located in the center of the Chongqing Dabashan National Nature Reserve. The aim of the nature reserve is to protect the subtropical forest ecosystems and their biodiversity.

**FIGURE 1:**
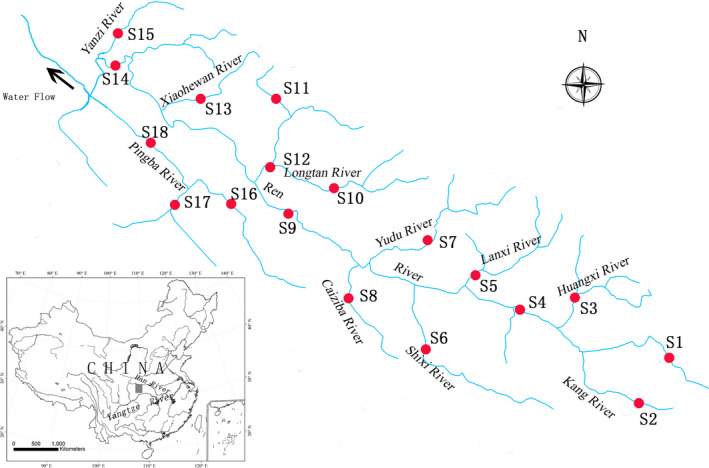
Sketch map of the sampling sites in the upper Ren River

In this study, fish fauna and habitat features were investigated in the upper Ren River, along a 128‐km stretch ranging from the headwater to the confluence with Pingba River (Figure 1). There are many medium‐to‐small streams (e.g., Kang River, Huangxi River, Shixi River, Lanxi River, Caiziba River, Longtan River, and Yanzi River) flowing into this stretch. The climate is typically subtropical monsoon, with hot–wet summers and cold–dry winters. The annual average temperature is 13.8℃, and the monthly mean temperature changes from 24.8℃ (July) to approximately 2.4℃ (January). The average annual rainfall is 1,261.4 mm, but is unevenly distributed in time, with 68% of total annual precipitation occurring in May to September. The river habitat is characterized by mainly deep valleys, shallow and winding channels, and rapid water flow. As the headwaters and most tributaries originate from primeval forest with less human influence, water quality in this area is high, except for a few sites due to urban sewage and industrial wastewater.

### 2.2 Fish sampling

Fish surveys were conducted in May–July and December 2017, representing the rainy and dry seasons, respectively. Each survey lasted for about 2 weeks. A total of 18 sites were sampled, covering the length of the main channel of the Ren River and its main tributaries. Among them, eight sites were located at 1st‐order streams, six at 2nd‐order, five at 3rd‐order, and one at 4th‐order (Table 1). The sampling sites were selected based on accessibility, similarity in habitat types, and to maximize the diversity of habitat types (Araújo et al., 2009). Fish specimens were caught by a backpack electrofisher (Susan 1030S, China; 12 V import, 250 V export) by a single pass (Liu et al., 2021). Each sampling was carried out at different habitat types (riffles, glides, and pools) by two people against the current. The length of each segment varied between 100 and 200 m, according to accessibility. After sampling, all captured specimens were identified to species level, measured (mm), and weighted (g). Individuals that could be confidently identified were released downstream from the sites after recovery, while individuals that could not be identified in the field were fixed in buffered formaldehyde (7%) and then transported to the laboratory for further taxonomic determination.

**TABLE 1:** Spatial parameters of sampling sites in mountain streams of the Ren River

Site code	Latitude	Longitude	Altitude (m)	S‐order	Link	C‐Link	D‐Link
S1	E109°11′07.1″	N31°47′35.6″	1,274	1	1	13	2
S2	E109°07′02.1″	N31°43′30.7″	1,290	1	1	13	2
S3	E109°04′15.5″	N31°48′32.3″	1,074	2	3	11	5
S4	E109°01′56.0″	N31°49′02.3″	991	3	5	11	6
S5	E108°56′07.2″	N31°51′37.1″	891	2	3	9	9
S6	E108°51′47.3″	N31°48′52.6″	899	1	1	8	10
S7	E108°52′26.1″	N31°53′50.1″	1,022	2	2	7	12
S8	E108°48′49.2″	N31°47′46.9″	1,040	1	1	6	13
S9	E108°42′20.3″	N31°55′07.7″	755	3	13	6	17
S10	E108°45′51.4″	N31°57′57.1″	1,083	2	2	6	4
S11	E108°40′12.4″	N32°03′48.1″	1,252	1	1	6	2
S12	E108°39′02.2″	N31°58′56.7″	777	3	4	5	21
S13	E108°31′11.3″	N32°03′51.2″	654	1	1	4	22
S14	E108°25′42.6″	N32°06′24.4″	456	4	20	3	25
S15	E108°28′09.7″	N32°10′07.2″	947	1	1	2	29
S16	E108°33′49.3″	N31°58′29.6″	652	1	1	2	3
S17	E108°32′11.9″	N31°57′34.1″	642	2	2	2	3
S18	E108°26′34.5″	N32°02′49.1″	470	2	3	1	30

For each site, habitat variables were measured prior to fish sampling. Specially, water temperature (℃), pH, dissolved oxygen (mg/L), and conductivity (μs/cm) were measured using an YSI6680 Multi Probe. Average channel width (m) was calculated using a Ranger Laser Finder instrument at the beginning, middle, and end of the sampling reach. Water depth was measured at three points (25%, 50%, and 75% of the transect width) along each transect. Current velocity (m/s) was determined in the middle of the sampling site with a LJD‐10 flow‐meter. Substratum composition was categorized into bedrock, cobble, pebble/gravel, sand, and mud/silt, according to Hoeinghaus et al. (2007). In addition, landscape variables were recorded in situ or calculated from topographical maps (Table 1). Latitude, longitude and altitude (m) were recorded by a Garmin GPS‐76 system at the time of field sampling. Stream order was assigned according to Strahler (1957). Description and calculation of stream link magnitude (link, Shreve, 1966), C‐link (Fairchild et al., 1998), and D‐link (Osborne & Wiley, 1992) were adapted from Smith and Kraft (2005). Measurements of these spatial parameters were referenced to a public map of Chengkou County (1:50 000).

### 2.3 Data analysis

Nonmetric multidimensional scaling (NMDS) ordination analysis, based on the Bray–Curtis similarity matrix (Clarke, 1993), was used to classify the spatial (stream order) and temporal (season) variations in the structure of fish assemblages. The Bray–Curtis similarity coefficient was calculated based on the relative abundance matrices. Rare species that occurred at less than three sites were excluded from the analysis. Next, one‐way analysis of similarity (ANOSIM) was carried out to determine whether fish assemblages changed significantly among stream orders or seasons. Then, a similarity of percentage analysis (SIMPER) was used to identify species that contributed most to the spatial or temporal dissimilarities of fish assemblages. All these analyses were performed with the PRIMER 5 software package (Clarke & Warwick, 2001), including modules “NMDS,” “SIMPER,” and “ANOSIM.”

Differences in environmental factors between seasons were tested by one‐way analysis of variance (ANOVA) using SPSS statistical programs (version 20.0). Relationships between fish assemblages and environmental factors for each season were examined by constrained canonical ordinations. Detrended correspondence analysis (DCA) was used to determine the appropriate model for direct gradient analysis (Leps & Smilauer, 2003). As the length of gradients for the first axis was estimated at 1.908 (<3) for the wet season and 1.746 (<3) for the dry season, redundancy analysis (RDA) was used in further analyses. Environmental variables that did not meet the normality assumption (Shapiro–Wilk test, *p* < .05) were transformed using natural logarithms, and collinear environmental variables with high variation inflation factors (VIF > 20) were eliminated from further analyses (McCune & Grace, 2002). Stepwise forward selection with Monte Carlo permutation tests (999 permutations, *p* < .05) was used to select a parsimonious set of explanatory variables. These analyses were performed using the software CANOCO for Windows 4.5 version (Ter Braak & Smilauer, 2002).

## 3 RESULTS

### 3.1 Species composition

A total of 1,330 individuals, represented by 21 fish species, were collected during the sampling period (Table 2). These species belonged to three orders, eight families, and 19 genera. Of these, *Scaphesthes macrolepis* has been listed the national Class II key protected wild animals of China, *Leptobotia hansuiensis* is endemic to the upper Han River drainage, and *S*. *macrolepis* and *Schizothorax prenanti* have been assessed as vulnerable by the China Species Red List (Jiang et al., 2016). Cypriniformes was the most abundant order, represented by 17 species and accounted for 91.0% of the total species, followed by Perciformes (three species) and Siluriformes (one species). Cyprinidae contained the highest abundance of families, represented by 13 species, and accounted for 61.9% of the total species. The remaining families comprised only one to three species.

**TABLE 2:** Total number (*N*), percent number (*N*%), weight (*W*, g), percent weight (W%), and percent frequency of occurrence of fish species in mountain streams of the Ren River

Species	Number (*N*)	*N* %	Weight (*W*)	*W* %	FO %
Cyprinidae					
*Zacco platypus*	70	5.26	341.2	4.11	22.64
*Opsariichthys bidens*	5	0.38	47.3	0.57	5.66
*Rhynchocypris oxycephalus*	669	50.30	2,484.3	29.90	71.70
*Hemiculter leucisculus*	57	4.29	683.0	8.22	3.77
*Hemibarbus labeo*	18	1.35	19.0	19.04	5.66
*Hemibarbus maculatus*	1	0.08	0.9	0.01	1.89
*Gnathopogon herzensteini*	128	9.62	751.9	9.05	41.51
*Squalidus argentatus*	47	3.53	311.3	3.75	9.43
*Platysmacheilus nudiventris*	4	0.30	27.8	0.33	3.77
*Pseudogobio vaillanti*	7	0.53	152.1	1.83	1.89
*Scaphesthes macrolepis*	113	8.50	1,279.8	15.40	30.19
*Spinibarbus sinensis*	3	0.23	2.4	0.03	1.89
*Schizothorax prenanti*	3	0.23	144.9	1.74	3.77
Noemacheilidae					
*Paracobitis ariegates*	6	0.45	29.8	0.35	7.55
Botiidae					
*Leptobotia hansuiensis*	14	1.05	29.4	0.35	3.77
Cobitidae					
*Cobitis sinensis*	20	1.50	84.3	1.01	15.09
Balitoridae					
*Metahomaloptera omeiensis*	125	9.40	152.6	1.84	43.40
Bagridae					
*Leiocassis crassilabris*	3	0.23	26.2	0.32	3.77
Percichthyidae					
*Siniperca scherzeri*	9	0.68	137.1	1.65	3.77
Gobiidae					
*Rhinogobius giurinus*	24	1.80	38.5	0.42	13.21
*Rhinogobius cliffordpopei*	4	0.30	5.3	0.06	3.77

The most common and abundant species was *Rhynchocypris oxycephalus*, which occurred in 71.7% of the sampling occasions and accounted for 50.3% of the total number of specimens captured. The next most abundant species was *Gnathopogon herzensteini*, which accounted for 9.6% the total number of specimens, followed by *Metahomaloptera omeiensis* (9.4%), *S*. *macrolepis* (8.5%), and *Zacco platypus* (5.3%). Some species, such as *Hemibarbus maculatus*, *Pseudogobio vaillanti*, *Spinibarbus sinensis,* and *S*. *prenanti*, occurred only occasionally.

### 3.2 Spatial and temporal variations in the structure of fish assemblages

**FIGURE 2:**
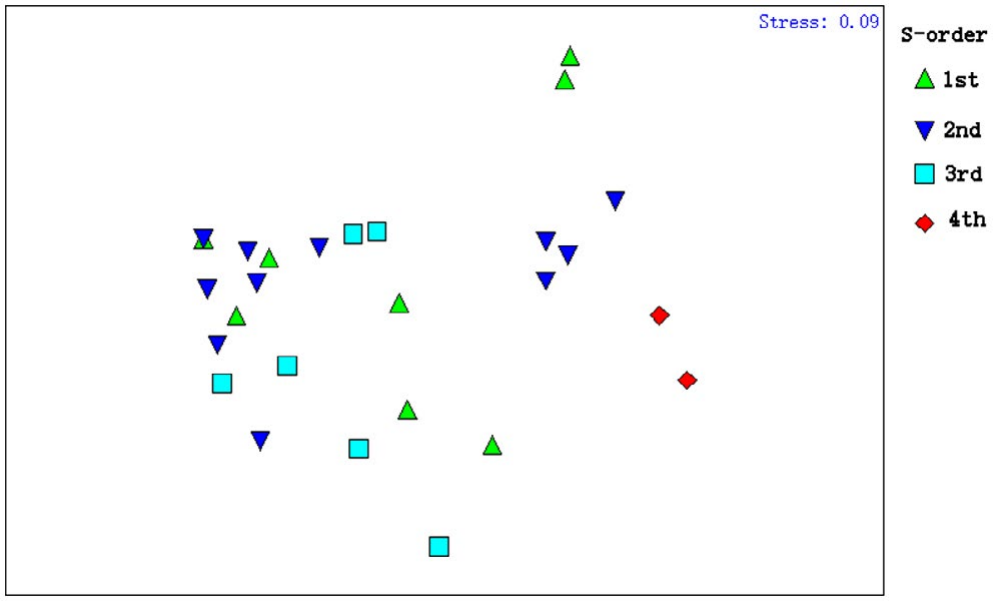
NMDS ordination plot of spatial (stream orders) changes in fish assemblage structure in mountain streams of the Ren River based on the Bray–Curtis similarity of fish species

The structure of fish assemblages varied significantly at the spatial scale. The NMDS ordination plot showed that sampling sites at the 3rd‐order streams were mainly gathered to the lower left of the graph, while those of the 4th‐order sites were clustered to the right (Figure 2). ANOSIM confirmed that the fish assemblages differed significantly among stream orders (Global *R* = 0.156, *p* = .034 < .05). According to the SIMPER analysis, fish assemblages in the Ren River basin were highly dominated by cold‐water or rheophilic species, such as *R*. *oxycephalus*, *S*. *macrolepi*, *M*. *omeiensis,* and *G*. *herzensteini*, except for the 4th‐order sites (Table 3). Specially, fish assemblages in 1st‐order sites were typified by *R*. *oxycephalus*, contributing 95.7% of the similarity. Fish assemblages in 2nd‐order sites were typified by *R*. *oxycephalus* and *G*. *herzensteini*, contributing 78.3% and 7.1% of the similarity, respectively. Fish assemblages in 3rd‐order sites were typified by *R*. *oxycephalus*, *M*. *omeiensis,* and *S*. *macrolepi*, contributing 55.4%, 28.1%, and 9.3% of the similarity, respectively. From 1st‐order sites to 3rd‐order sites, the abundance of *R*. *oxycephalus* decreased gradually, while the abundance of *M*. *omeiensis* and *S*. *macrolepi* increased continuously. By contrast, fish assemblages in 4th‐order sites were predominated mainly by warm‐water and lentic or eurytopic species, such as *Squalidus argentatus*, *Hemiculter leucisculus,* and *Z. platypus*.

**TABLE 3:** Typifying species determined by SIMPER analysis for fish assemblages in different stream orders in the Ren River

Stream order	Species	Average abundance (%)	Average similarity (%)	Contribution %
1st	*P. oxycephalus*	69.5	47.3	95.7
2nd	*P. oxycephalus*	53.8	28.3	78.3
	*G. herzensteini*	15.4	2.6	7.1
3rd	*P. oxycephalus*	35.2	22.4	55.4
	*M. omeiensis*	24.0	22.1	28.1
	*S. macrolepis*	16.4	3.8	9.3
4th	*S. argentatus*	27.1	26.4	54.1
	*H.leucisculus*	3.6	9.4	19.3
	*Z. platypus*	7.5	5.6	11.4
	*G. herzensteini*	20.5	3.3	6.8

Note

Only species that contribute to >5% of the average similarity within group are shown.

**FIGURE 3:**
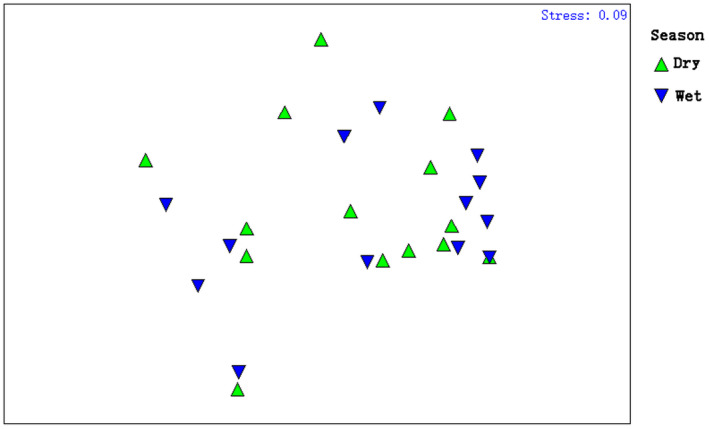
NMDS ordination plot of seasonal changes of fish assemblage structure in mountain streams of the Ren River based on the Bray–Curtis similarity of fish species

There was no significant seasonal change in fish assemblages. NMDS ordination plots revealed substantial overlap between fish assemblages in wet and dry seasons (Figure 3). ANOSIM further confirmed that the fish assemblages did not show significant changes across seasons (Global *R* = −0.022, *p* = .745 > .05).

### 3.3 Relationships between fish assemblages and environmental factors

The altitude of the 18 sampling sites ranged from 456 m to 1,290 m. Stream orders ranged from 1st to 4th. Link, C‐link, and D‐link ranged from 1 to 20, 1 to 13, and 2 to 30, respectively (Table 1). ANOVA showed that water temperature, pH, dissolved oxygen, conductivity, velocity, and the percent of cobble varied significantly with seasons (Table 4). In particular, water temperature in the wet season was significantly higher than that in dry season, while pH, dissolved oxygen, conductivity, velocity, and the percent of cobble showed the opposite trend. Other variables showed no significant seasonal changes.

**TABLE 4:** Mean ± *SE* values of different habitat variables at 18 sites in the Ren River

Habitat variables	Wet season	Dry season	*F‐*ratio	*p*‐value
Water temperature (℃)	23.3 ± 2.2	12.1 ± 1.4	4,962.836	.000
pH	7.5 ± 0.7	9.0 ± 0.3	627.012	.000
Dissolved oxygen (mg/s)	7.7 ± 1.0	10.4 ± 1.4	420.589	.000
Conductivity (μs/cm)	244.8 ± 72.7	269.7 ± 107.6	385.900	.000
Velocity (m/s)	0.8 ± 0.3	0.5 ± 0.2	5.603	.028
width (m)	5.4 ± 3.2	4.4 ± 2.9	3.830	.052
depth (m)	0.7 ± 0.3	0.5 ± 0.2	3.916	.063
Bedrock%	43.9 ± 14.6	42.5 ± 15.7	0.586	.444
Cobble%	28.6 ± 7.4	30.3 ± 9.2	28.048	.000
Pebble%	20.0 ± 9.5	19.7 ± 9.5	0.120	.729
Sand%	4.2 ± 4.6	7.2 ± 4.3	0.355	.552
Silt %	0.4 ± 0.3	0.3 ± 1.2	0.295	.593

After the forward selection procedure, three environmental variables (altitude, C‐link, and channel width) were finally retained for the wet season, while five environmental variables (altitude, C‐link, channel width, water temperature, and depth) were retained for the dry season.

For the wet season, altitude explained the most variance (44.5%), followed by C‐link (33.6%) and width (15.9%). The species–environment correlations of the first two ordination axes were 0.924 and 0.526, respectively. The first ordination axis accounted for 45.6% of the variance of the species data, while the second axis accounted for 50.6% of this variance. Altitude (−0.911) and C‐link (−0.779) were negatively correlated with the first axis, while channel width (0.529) was positively correlated.

For the dry season, altitude explained the most variance (37.1%), followed by C‐link (24.0%), width (20.0%), water temperature (15.7%), and depth (14.7%). The species–environment correlations of the first two ordination axes were 0.946 and 0.591, respectively. The first ordination axis accounted for 39.6% of the variance of the species data, which the second axis accounted for 46.3% of this variance. Altitude (−0.915) and C‐link (−0.709) were negatively correlated with the first axis, while channel width (0.620), water temperature (0.587), and depth (0.514) were positively correlated.

**FIGURE 4:**
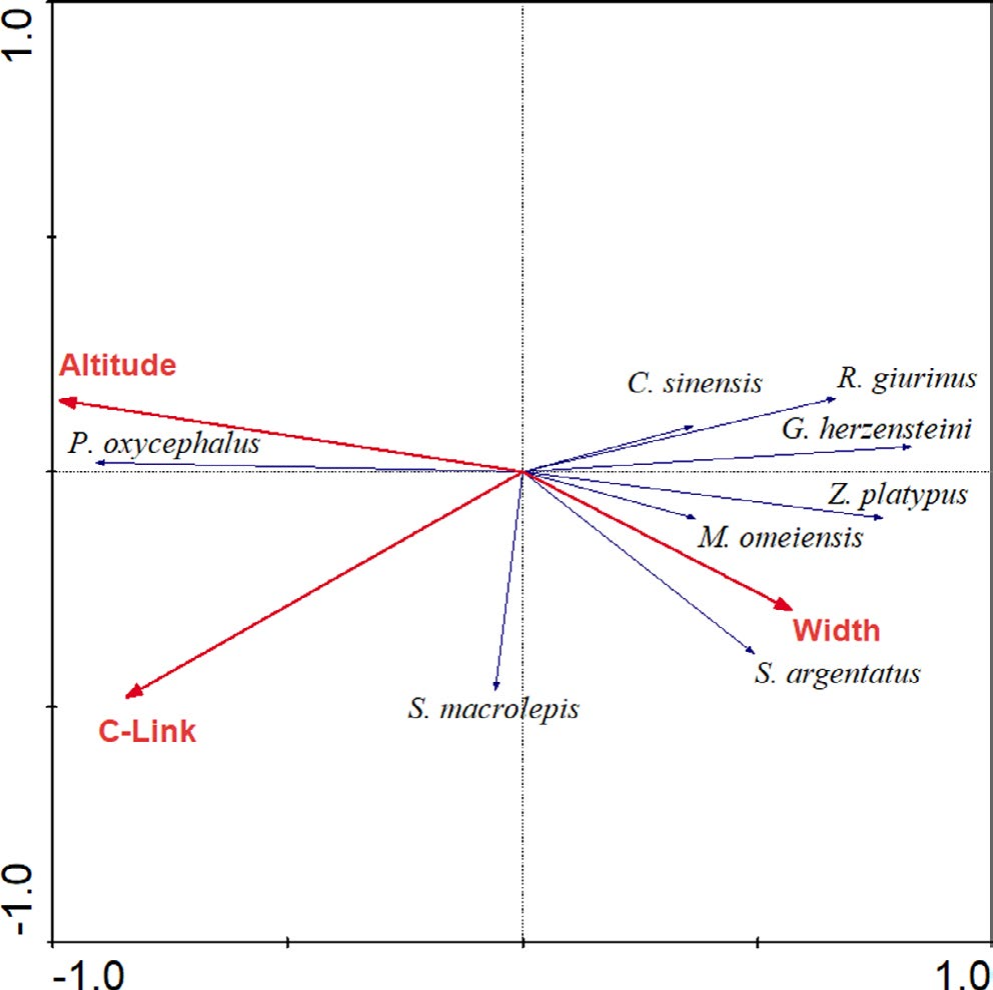
RDA ordination plots showing relationship between fish assemblages and environmental variables in the Ren River basin during the wet season

**FIGURE 5:**
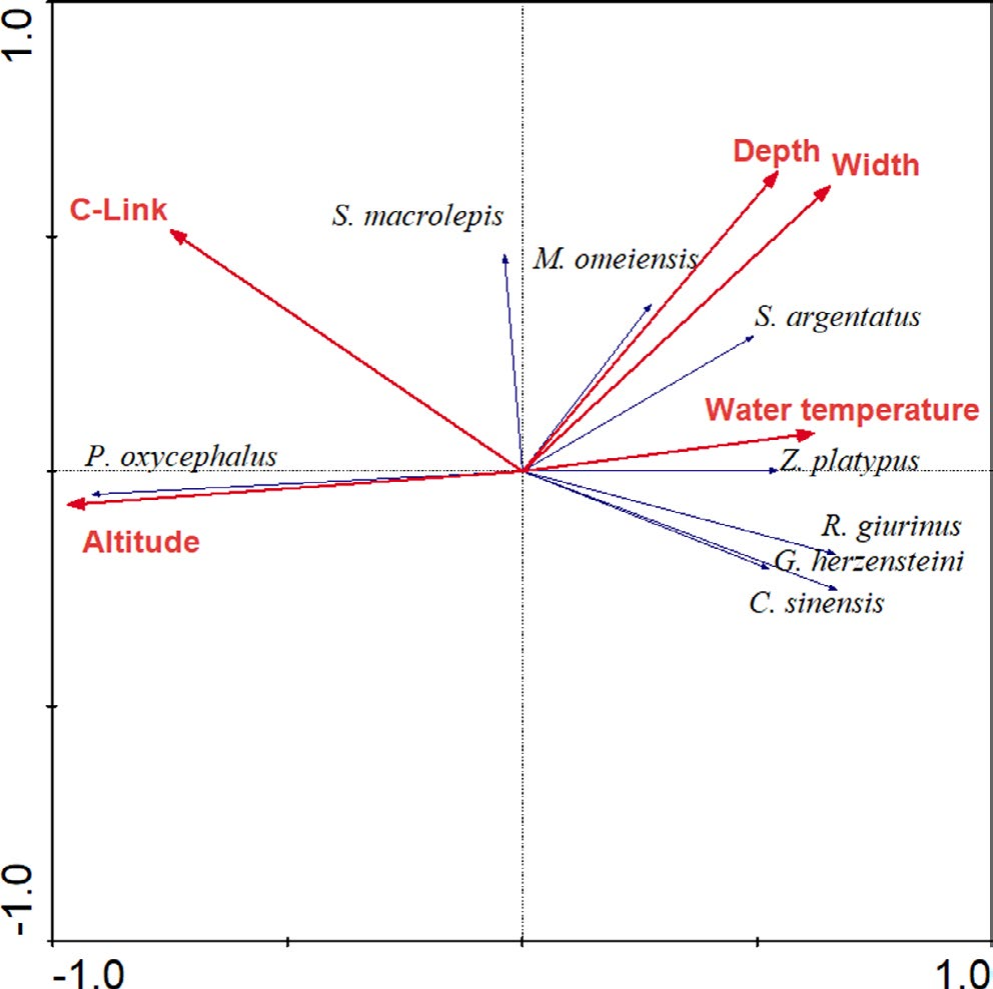
RDA ordination plots showing relationship between fish assemblages and environmental variables in the Ren River basin during the dry season

The RDA ordination plots showed that *R*. *oxycephalus* was positively related to altitude and negatively related to channel width in both seasons (Figures 4 and 5). *S*. *macrolepis* was positively related to C‐link in both seasons. However, the species–environment association of other species, such as *S*. *argentatus*, *Z. platypus*, *G*. *herzensteini*, *M*. *omeiensis*, *Cobitis sinensis,* and *Rhinogobius giurinus*, showed some changes across seasons. In the wet season, all these species were positively related to channel width (Figure 4). In the dry season, however, some species (e.g., *Z. platypus*, *G*. *herzensteini*, *C*. *sinensis,* and *R*. *giurinus*) preferred habitats with high water temperature and some species (e.g., *M*. *omeiensis*) tended to occupy deep water (Figure 5).

## 4 DISCUSSION

### 4.1 Spatial and temporal patterns of fish assemblages

Several studies have observed significant spatial and temporal variations in fish assemblages in natural streams (Fernandes et al., 2013; He et al., 2017; Silvano et al., 2000). In particular, spatial variations in fish assemblage were usually caused by changes in habitat features along the longitudinal gradient, while seasonal variability in fish assemblages was often attributed to flood‐related changes in habitat characteristics and induced seasonal migration of fish species (Fernandes et al., 2013; He et al., 2017; Silvano et al., 2000).

The present study revealed that fish assemblages in streams of the Ren River basin varied significantly with stream order. However, no significant changes in fish assemblages were observed between seasons. These results are consistent with some other studies (Fernandes et al., 2013; Habit et al., 2007; Li et al., 2012; Yan et al., 2010; Zhu et al., 2017). For example, Ostrand and Wilde (2002) found spatial components of variation in fish assemblages in the upper Brazos River were greater than seasonal components. Fernandes et al. (2013) revealed that fish assemblages in Meridional Amazonian streams changed among watersheds (spatial variation), but not seasonally (temporal variation). Fish assemblages in Northwestern Great Plains streams varied more spatially than temporally (Mullen et al., 2011). These streams were in the United States. Similar findings were also observed in the lower Yangtze River, China (Li et al., 2012; Yan et al., 2010; Zhu et al., 2017). Yan et al. (2010) observed that fish assemblages in the Puxi Stream were significantly different in spatial variation but not in temporal variation. Li et al. (2012) found that fish assemblages in a mountain stream of the north Tiaoxi River differed along the stream continuum, but there was little apparent change associated with the seasons. These studies together suggest that seasonal variations in habitat features may not always lead to temporal changes in the structure of fish assemblages. One of the possible reasons is that fish assemblages in these stream systems were determined more by average or persistent spatial heterogeneity in environmental conditions and environmental variability than by seasonal variation in environmental conditions (Li et al., 2012; Mullen et al., 2011; Ostrand & Wilde, 2002).

In this study, some habitat variables (e.g., water temperature, pH, dissolved oxygen, conductivity, velocity, and substrate composition) changed markedly from the wet season to the dry season. However, temporal changes in these habitat variables were probably exceeded by deterministic catchment geomorphology and climate along the longitudinal gradient. The latter was emphasized by the Riverine Ecosystem Synthesis (Thorp et al., 2006). As a result, fish assemblages were determined mainly by large‐scale spatial variables (e.g., altitude and C‐link), while local habitat variables played less important roles or just acted in a single season. In addition, fish assemblages in the Ren River basin were dominated mainly by resident species, such as *R*. *oxycephalus*, *G*. *herzensteini*, *M*. *omeiensis*, *S*. *macrolepis,* and *Z. platypus*. These species were well‐adapted to the instream running water and did not depend on long‐distance migration to complete their life cycles. Therefore, the lack of seasonal change in fish assemblages in streams of the Ren River probably resulted from the natural variability of the river system and the sedentary life habit of fish species.

### 4.2 Environmental effects on fish assemblages

The present study showed fish assemblages in streams of the Ren River basin were structured by a combination of large‐scale landscape factors (e.g., altitude and C‐link) and local habitat features (e.g., channel width, water temperature, and depth), among which, landscape factors acted as the most important contributor in both wet and dry seasons. The importance of landscape position in structuring fish assemblages has been demonstrated by numerous studies (e.g., Fairchild et al., 1998; He et al., 2017; Li et al., 2012; Smith & Kraft, 2005; Zhu et al., 2017). With regard to landscapes therefore, streams should be regarded as connected networks with a definable “network geometry,” rather than a linear hierarchy most represented by stream order (Benda et al., 2004; Smith & Kraft, 2005). Abiotic and biotic stream characteristics change from low‐order headwater streams to high‐order downstream locations (Fausch et al., 1984; Smith & Kraft, 2005). Specifically, high altitude and low‐order stream often have low water temperature, narrow channels, shallow water, and low immigration (Murugavel & Pandian, 2000). Therefore, landscape position in stream network geometry can influence the distribution patterns of fish species and thus assemblage structure (Li et al., 2012; Smith & Kraft, 2005). In this study, *R*. *oxycephalus*, a typical cold‐water species, was restricted to headwaters and small tributaries with higher elevation and absent from the lower locations. By contrast, species that are adapted to warm‐water and a lentic environment (e.g., *S*. *argentatus* and *H*. *leucisculus*) were distributed exclusively in the lower locations. The barrier caused by the extreme and harsh environmental conditions in high altitudes seems to have effectively prevented the dispersion and colonization of these fish species from the lowlands (Li et al., 2012).

Compared to the abovementioned landscape spatial factors, local habitat factors (e.g., channel width, water temperature, and depth) played less important roles in determining fish assemblages in the Ren River basin. However, the influence of these factors increased somewhat in the dry season. In the wet season, water temperature and resource availability were not the limiting factors for warm‐water fish species, such as *S*. *argentatus*, *H*. *leucisculus,* and *Z. platypus*, because they were high enough to satisfy the ecological requirements of these species. However, as the water level and water temperature declined in winter, diversity, and availability of habitat and food resources became limited (Liu et al., 2019). In this situation, these species tended to occupy larger and deeper habitats with warmer water temperatures and richer food resources (Li et al., 2012).

### 4.3 Implications for conservation

This study is the first to describe the spatial–temporal variation of fish assemblages in mountain streams in the southwest China. Information obtained from this study will enhance our understanding of the variation in fish assemblages and then help to develop strategies for future fish diversity protection and conservation in such mountain streams. A total of 21 fish species were collected during the sampling period. Among these, *S*. *macrolepis* and *S*. *prenanti* have been considered as vulnerable. These species used to be important commercial targets in the Ren River basin. However, compared with historic records, their distribution range has shrunk significantly, and their population size has seriously declined (Zeng, 1991). In addition, the miniaturization tendency of these two species has become evident (Zeng, 1991). The prospects of survival of *S*. *prenanti* are gloomier, as only three specimens were collected during our study. Overfishing might be the main reason responsible for this phenomenon (Zeng, 1991). To protect these vulnerable species, the local government should strengthen fishery management and fight against illegal fishing. In addition, the present study indicated that fish assemblages in streams of the Ren River basin were influenced mainly by landscape factors, such as altitude and C‐link. Specially, *R*. *oxycephalus*, a representative cold‐water species, was restricted to headwaters and small tributaries with higher elevation. This species is an excellent bioindicator species for low‐temperature water and acts as a good model species for studying freshwater fish biogeography, because of its low dispersal ability and restriction to small mountain habitats (Yu et al., 2014). Climate change and anthropogenic interferences have seriously threatened this species (Yu et al., 2014). Therefore, to maintain the stability of fish assemblages' structure in such stream ecosystems, human activities that will destroy the naturality and connectivity of stream habitat, such as deforestation, dredging, and damming, should be strictly forbidden.

## CONFLICT OF INTEREST

None declared.

## AUTHOR CONTRIBUTIONS

**Fei Liu:** Conceptualization (lead); data curation (lead); formal analysis (lead); investigation (lead); methodology (lead); resources (lead); software (lead); supervision (lead); writing‐original draft (lead). **Pengcheng Lin:** Data curation (supporting); investigation (supporting); methodology (supporting); writing‐original draft (supporting). **Huanzhang Liu:** Funding acquisition (lead); project administration (lead); writing‐review & editing (equal). **Jun Wang:** Investigation (supporting); methodology (supporting); visualization (supporting); writing‐original draft (supporting).

## Data Availability

Fish and environmental variable data were deposited in the Dryad Digital Repository, https://doi.org/10.5061/dryad.jq2bvq886. Other relevant data can be accessed in the manuscripts.
